# Drug prescription by telephone consultation in Danish out-of-hours primary care: a population-based study of frequency and associations with clinical severity and diagnosis

**DOI:** 10.1186/1471-2296-15-142

**Published:** 2014-08-20

**Authors:** Grete Moth, Linda Huibers, Morten Bondo Christensen, Peter Vedsted

**Affiliations:** 1 Research Unit for General Practice, Department of Public Health, Aarhus University, Bartholins Alle 2, Aarhus 8000, Denmark

**Keywords:** Denmark, General practice, After-hours care, Prescriptions, Telephone

## Abstract

**Background:**

Danish general practitioners (GPs) answer all calls to the out-of-hours primary care service. About 60% of the calls are terminated on the telephone through provision of medical advice and prescription of medication. Nevertheless, little is known about the prescription patterns of telephone consultations, such as prescription frequency and indications for drug use. Our aim was to examine the characteristics of patients and GPs in telephone consultations resulting in drug prescription.

**Methods:**

The study was based on a 12-month survey on reasons for encounter in the Danish out-of-hours primary care service. A total of 385 GPs (55.5% of all GPs from Central Denmark Region on duty during a year) participated in answering electronic pop-up questionnaires integrated in the electronic patient administration system. The questionnaires contained items on reasons for encounter (e.g. existing chronic disease or new health problem), diagnoses, and GP-assessed severity of the health problem. Data on time of contact, patient gender and age, and prescribed medication (Anatomic Therapeutic Chemical classifications) for telephone consultations were obtained from the patient administration system. Differences in characteristics of patients, general practitioners, and contacts were examined, and associations with prescribed medication were analysed using a multivariate analysis with prevalence ratios.

**Results:**

Medication was prescribed in 19.9% of the included 4,173 telephone consultations; antibiotics and analgesics were prescribed most frequently (10.8% and 2.5%, respectively). GPs tended to assess contacts resulting in antibiotic prescription as more severe than other contacts. For high-severity contacts, there was a lower likelihood for prescription (prevalence ratio = 0.28 (0.16-0.47)). Children aged 0-4 years had lower probability of receiving a prescription compared with patients aged 18-40 years. The prescription rate was highest during the first four hours of the opening hours of the out-of-hours primary care service.

**Conclusion:**

One in five of all telephone consultations involved drug prescription; antibiotics constituted half of these prescriptions. Drug prescription by telephone was less likely to be offered in cases involving ‘severe’ reason for encounter or children. This study calls for further studies of drug prescriptions issued via out-of-hours primary care telephone consultations.

## Background

Out-of-hours primary care service (OOH-PC) is an important component in a health-care system covering the public need for medical advice and help during the time outside office hours. Growing patient demands [1-3] in combination with increasing needs of diagnosis and treatment outside normal working hours place heavy demands on the modern healthcare system; these challenges have created a constant need for further development of the organisation of OOH-PC [4-6]. In Denmark, a reform in 1992 aimed to place general practitioners (GPs) in the first line of care by making them responsible for answering and triaging telephone calls from all patients [6,7]. The Danish model with GPs answering calls in the frontline is different from the OOH-PC organisation in most other countries, where nurses, assistants or other health care personnel perform the telephone triage [8]. The Danish GPs can end the triage contact by telephone and prescribe medication (if needed), or they can schedule a follow-up clinic consultation or home visit.

Telephone triage in OOH-PC is used as a tool to manage patient flows efficiently and safely [8,9]. A relatively large share of calls (59%) to the Danish OOH-PC is terminated by telephone with medical advice or direct referral to the hospital in acute cases [10]. The GP’s medical advice in a telephone consultation may include prescription of medication. Due to the acute nature of patient calls to OOH-PC, a need for medication can be expected. The appropriateness and extent of medication prescription by telephone in OOH primary care are important issues, which are constantly subject for debate. Nevertheless, knowledge on prescriptions in the OOH-PC telephone consultations is sparse. Another challenge is that the OOH-PC may be used for convenience when the daytime GP is not available or when a repeated prescription is needed. Thus, it may be possible to increase the efficiency of prescriptions in the OOH-PC.

The aim of this study was to examine patient, GP, and contact characteristics of telephone consultations involving drug prescription and to analyse factors associated with prescription of drugs.

## Methods

### Design, setting, and data collection

We performed a cross-sectional population-based study on prescriptions in the OOH-PC in the Central Denmark Region, comprising 1.2 million people and corresponding to 22.7% of all Danish inhabitants [11]. The OOH-PC organisation provides primary care and is staffed by fully licensed GPs (and some GP trainees) in a tax-funded fee-for-service system to which all citizens have free access. The electronic patient administration system is fully computerised, implying that the medical records of prescriptions and contacts are valid and complete.

We combined data from the patient administration system with collected data from the LV-KOS study, an extensive observational survey on reasons for encounter (RFE) and disease patterns in the Danish OOH-PC [12]. In the LV-KOS study, GPs filled in a pop-up questionnaire that was integrated in the patient administration system and appeared after one in ten telephone contacts. The LV-KOS study was conducted from 1 June 2010 to 31 May 2011, and 385 GPs (55.5% of all GPs on duty during the year) participated at least once registering a total of 21,457 patient contacts [12]. As GPs participated a varying number of times (1-86 times) there was a large variation in the number of registered telephone consultations per GP (1-334). In the present study, we only included terminated telephone consultations where a treatment decision was made. That is, telephone contacts ending with referral to a subsequent face-to-face contact or hospital admission were excluded as the choice of treatment in these cases was left to another physician.

### Variables

Telephone consultations in the LV-KOS study included information on the GP-coded probable diagnosis classified by the International Classification of Primary Care (ICPC-2) [13] and GP-assessed severity of contact (‘Severe’, ‘Potentially severe and the patient needs to be seen’, ‘Not severe’, ‘Not ill’, and ‘Don’t know’). In addition, each call was registered as either due to a ‘new episode of illness’ or an ‘exacerbation of a chronic disease’. We also included data on patient age, gender, and time of contact from the patient administration system as well as GP age and gender. All prescriptions were automatically registered in the system using the Anatomical Therapeutic Chemical (ATC) Classification System [14].

As we focused exclusively on telephone consultations that were relevant for decision-making on treatment, we identified and excluded renewal of prescriptions on the basis of the GP registrations. Next, we identified the most frequently prescribed types of medication. As antibiotics are classified from more than one ATC main group, we gathered all relevant ATC codes from other ATC groups in one group (see Additional file 1). Likewise, as analgesics are frequently prescribed, we also formed one group of analgesics from various ATC main groups (Additional file 2). The classifications for these groups were defined by two medical researchers and reviewed by a third who was also a GP. We aggregated the less frequently prescribed medication into an ‘Other medication’ group.

All telephone consultations involving a prescription were classified into diagnostic subgroups according to the components of the ICPC-2 coding system (i.e. infections, injuries, and other diagnoses such as neoplasms, congenital anomalies, and specific diseases) [13]. Furthermore, we formed a diagnostic group consisting of the contacts for which the GP had stated a symptom as the diagnosis (using ICPC symptom codes), but not a final diagnosis.

The GP-assessed severity of the health problem was dichotomised into ‘Potentially severe’ (combining ‘Severe’ and ‘Potentially severe and the patient needs to be seen’) and ‘Not severe’ (combining ‘Not severe’ and ‘Not ill’), while ‘Don’t know’ was recoded as missing (4.5% of the cases). We constructed a variable, ‘Number of hours from office hours’, categorising the number of hours from the start of the OOH-PC opening hours until the time of the actual telephone contact (<5, 5-8, and > 8 hours; more than 8 hours applicable only in weekends and holidays).

### Analyses

Descriptive analyses of prescriptions were performed using Chi2 tests for estimating differences between patient groups, GPs, and contact characteristics. Consultations were stratified into groups according to patient gender and age (0-4, 5-17, 18-40, 41-60, and above 60 years). Likewise, Chi2-tests were used to compare the prescription groups within the diagnosis groups, the severity groups and within the categories based on whether the contacts were new episodes or due to chronic disease. Moreover, associations between having an OOH-PC telephone consultation and having medicine prescribed were calculated by use of a multivariate regression analysis in the form of a generalised linear model (GLM) adjusted for patient gender and age group, GP-assessed severity of health problems, GP gender and age group, and number of hours from opening hours. Moreover, as the GPs registered more than one contact we adjusted for clustering to meet the assumption of independence between observations (robust variance estimate). We preferred the prevalence ratio (PR) to the odds ratio (OR) because the prevalence was high (>20%) and the ORs, therefore, would tend to overestimate the PR [15]. Analyses were performed with STATA 13.

### Ethics and approvals

The LV-KOS project was approved by the Danish Data Protection Agency (J.no. 2009-41-4069) and by the Danish Health and Medicines Authority (J.no. 7-604-04-2/122/EHE). According to Danish law, approval from the ethical committee was not needed as no biomedical intervention was performed, and the study did not present any ethical problems.

## Results

A total of 4,354 telephone consultations were registered. The renewal of a prescription was the RFE in 181 (4.2%) of the telephone consultations; these calls were excluded from the analyses leaving 4,173 contacts. Medications concerning the nervous system (primarily antidepressants and antipsychotics) and the respiratory system were the most frequently renewed prescriptions (24.9% and 14.9%, respectively) (data not shown).

### Frequency of prescriptions

In 19.9% of all telephone consultations, a prescription was made (Table 1); medications were most often prescribed to the age groups 18-40 years (22.6%; 95% CI: 20.5-24.9) and 41-60 years (23.4%; CI: 20.4-26.9). In 8.3% of the consultations resulting in prescriptions, two or more prescriptions were made (data not shown). No differences in prescribing patterns were associated with GP characteristics. When the GPs assessed the RFE as ‘potentially severe’, fewer patients were prescribed medication (6.3%; CI: 4.1-9.5 vs. 21.3%; CI: 20.0-22.6).

**Table 1:** **Patient and GP characteristics of 4377 telephone consultations**^**1**^**and drug prescription rates**

	**Telephone consultations N (%)**	**Prescription rates % (95% CI)**
**All contacts**	4,377 (100)	19.9 (17.7-20.0)
**Patient characteristics**		
**Gender**		
Female	2,285 (54.8)	21.5 (19.9-23.2)
Male	1,888 (45.2)	18.0 (16.3-19.8)
**Age**		
0-4 years	841 (20.25)	15.2 (13.0-17.8)
5-17 years	563 (13.5)	17.2 (14.3-20.6)
18-40 years	1,398 (33.5)	22.6 (20.5-24.9)
41-60 years	702 (16.8)	23.4 (20.4-26.6)
>60 years	669 (16.0)	18.7 (15.9-21.8)
**GP characteristics**		
**Gender**		
Female	1,140 (27.3)	17.9 (15.8-22.1)
Male	3,033 (72.7)	20.6 (19.2-22.1)
**Age**		
<41	1,349 (32.3)	19.2 (17.2-21.4)
41-50	879 (21.1)	18.2 (15.8-20.9)
51-60	1,176 (28.2)	21.9 (19.6-24.3)
>60	769 (18.4)	20.0 (17.3-23.0)
**Contact characteristics**		
**GP-assessed severity**		
Severe/potentially severe	320 (7.7)	6.3 (4.1-9.5)
Not severe/not ill	3,642 (87.3)	21.3 (20.0-22.6)
Missing	211 (5.1)	17.1 (12.6-22.8)
**New episode/chronic disease**		
New episode	3,402 (81.5)	19.6 (18.3-21.0)
Exacerbation of chronic disease	492 (11.8)	18.5 (15.3-22.2)
Missing/not relevant	279 (6.7)	26.2 (21.3-31.7)
**Number of hours from office hours**		
<5	806 (19.3)	19.1 (16.5-22.0)
5-8	494 (11.8)	11.9 (9.4-15.1)
>8 (weekends)	2,873 (68.9)	21.5 (20.0-23.0)

^1^Prescription renewals excluded.

**Figure 1:**
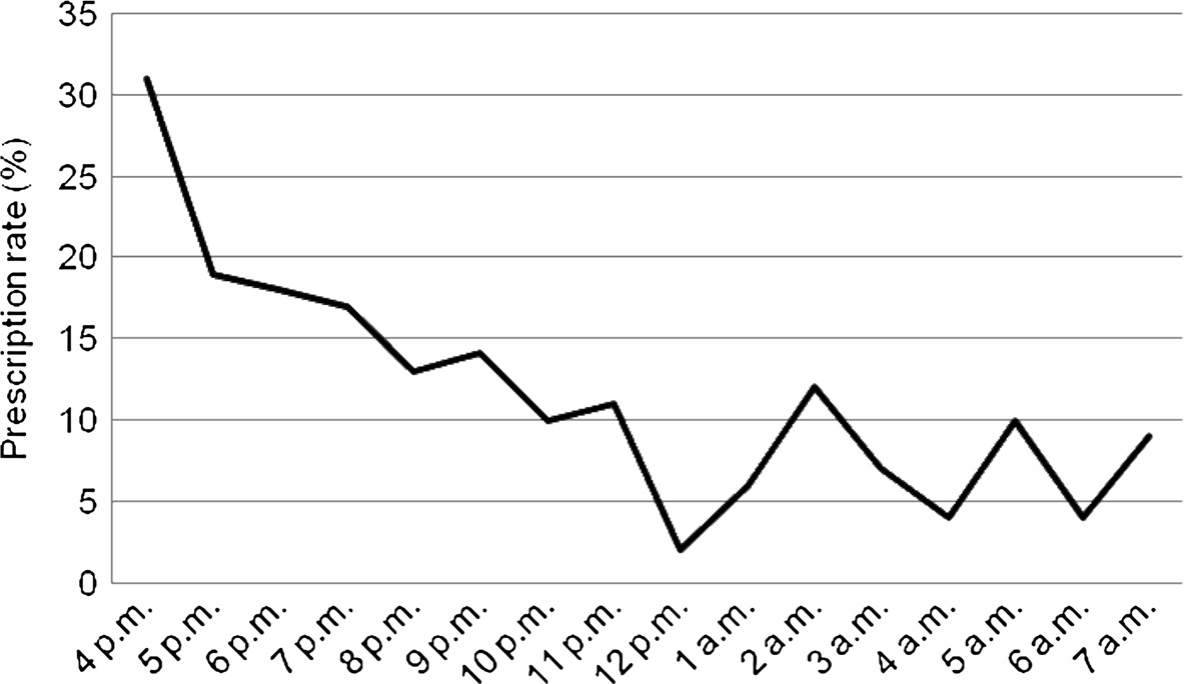
Prescription rate during OOH opening hours on weekdays (4 pm – 8 am).

**Figure 2:**
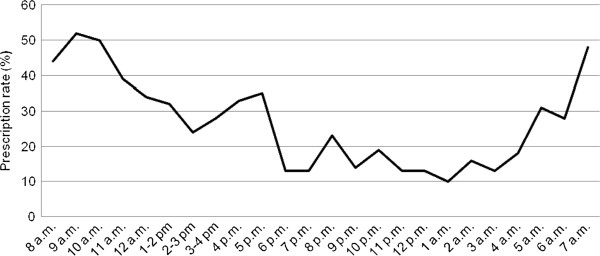
Prescription rate during OOH opening hours on weekends and public holidays.

The prescription rate peaked during the first opening hours of the OOH-PC on weekdays and from early morning and through the daytime during weekends and public holidays (Table 1 and Figures 1 and 2). The highest prescription rate was found in the morning during weekends and public holidays (Figure 2).

Antibiotics were the most often prescribed type of medication (10.8%) in telephone consultations (Table 2) and were prescribed in 54.1% of all contacts resulting in prescriptions. In consultations with no final diagnosis, analgesics were most often prescribed (3.8%). Antibiotics were more often prescribed in case of a new episode of illness (10.9%) as compared with exacerbations of chronic disease (6.4%). Health problems involving prescription of antibiotics were more often assessed as ‘potentially severe’ by the GPs than health problems involving prescriptions of other medications (2.5% vs. 0.2-1.0%).

**Table 2:** **Prescription rates per type of medication**^**1,2**^**stratified for diagnosis and severity of telephone consultations**

	**Contacts without prescription N (%)**	**Antibiotics N (%)**	**Analgesics N (%)**	**Medication in respiratory system N (%)**	**Other prescriptions N (%)**	**p-value**^**3**^
**Number of contacts** (4,173 in total)	3,343 (80.1)	451 (10.8)	103 (2.5)	66 (1.6)	213 (5.1)	
**Diagnosis groups**						
- Symptoms (no disease diagnosis applied) (1,021)	916 (90.1)	22 (2.1)	40 (3.8)	6 (0.6)	38 (3.6)	<0.001
- Infections (1,335)	908 (68.3)	367 (27.2)	8 (1.0)	14 (1.0)	40 (3.0)	
- Injuries (770)	712 (92.9)	23 (2.8)	22 (1.9)	4 (0.5)	9 (1.1)	
- Other diagnoses (1,047)	807 (79.0)	39 (3.5)	33 (2.9)	42 (3.6)	126 (11.0)	
**Severity** (missing = 211)						
- Severe/potentially severe (320)	300 (95.8)	12 (2.5)	5 (1.0)	1 (0.2)	2 (0.6)	0.185
- Not severe/not ill (3,642)	2,868 (78.7)	421 (11.6)	90 (2.5)	63 (1.7)	203 (5.6)	
**New episode or chronic disease** (missing = 279)						
- New episode (3,402)	2,736 (81.4)	391 (10.9)	71 (2.0)	52 (1.5)	154 (4.3)	<0.001
- Exacerbation of chronic disease (492)	401 (82.4)	33 (6.4)	25 (4.8)	10 (1.9)	24 (4.6)	

^1^Prescriptions sums add up to more than the total number of contacts as some contacts resulted in more than one prescription.

^2^Prescription renewals excluded.

^3^Test of difference between contact groups involving prescription of medication (using Fishers exact for severity due to few observations).

The PR of medication prescription was not associated with patient gender or GP characteristics (Table 3). Compared with adults aged 18-40, children below five years of were less likely to get a prescription. If the problem was assessed as ‘potentially severe’ by the GP, the propensity of receiving a prescription was lower (0.17; CI: 0.10-0.29) than if ‘not severe’. The probability of getting a prescription was higher within the first opening hours of the OOH-PC compared with the following four hours.

**Table 3:** Prevalence of prescriptions* based on telephone consultation and prevalence ratio (PR) adjusted for patient gender and age group, type of contact (new episode or exacerbation of chronic disease), GP gender and age group, and GP-assessed severity of health problem

	**Prescription frequency**	**Adjusted PR**	
	**(%)**	**(95% CI)**	**P-value**
All = 4,382*			
**Patient gender**			
Female	21.5	1 (ref.)	
Male	18.0	0.88 (0.78-1.00)	0.042
**Patient age**			
0-4 years	15.2	0.68 (0.56-0.83)	<0.001
5-17 years	17.2	0.81 (0.66-0.99)	0.040
18-40 years	22.6	1 (ref.)	
41-60 years	23.4	1.10 (0.91-1.32)	0.302
>60 years	18.7	0.89 (0.72-1.10)	0.267
**GP gender**			
Female GP	17.9	1 (ref.)	
Male GP	20.6	1.14 (0.86-1.49)	0.364
**GP age**			
<41 years	19.2	1 (ref.)	
41-50 years	18.2	1.00 (0.76-1.33)	0.990
51-60 years	21.9	1.09 (0.86-1.38)	0.498
>60 years	20.0	1.06 (0.69-1.61)	0.805
**GP-assessed severity**			
Not severe/not ill	21.3	1 (ref.)	
Severe/potentially severe	6.3	0.28 (0.16-0.47)	<0.001
**New episode or chronic disease**			
New episode	19.6	1 (ref.)	
Exacerbation of chronic disease	18.5	0.92 (0.74-1.15)	0.449
**Number of hours from office hours**			
1-4 hours	19.1	1 (ref.)	
5-8 hours	11.9	0.56 (0.44-0.72)	<0.001
>8 hours	21.5	1.10 (0.93-1.31)	0.278

^*^Prescription renewals excluded.

## Discussion

### Main findings

In this population-based survey on the Danish OOH-PC, 4% of all contacts concerned prescription renewals. Excluding these, we found that one in five of all OOH-PC telephone consultations involved medication prescription. The prescription rate was highest during the first opening hours immediately after the closure of daytime general practice. Antibiotics were most frequently prescribed, followed by analgesics and medication for the respiratory system. A large share of health problems involving antibiotic prescription were assessed as ‘potentially severe’ compared with health problems involving prescriptions of other medications. Children below five years of age were the least likely to a prescription. The propensity of receiving a prescription was highest in the weekend, while the next-highest was having a contact during the first opening hours of the OOH-PC. Antibiotics were more often prescribed for a new episode of illness compared to a consultation for a chronic disease.

### Strengths and limitations

The study was based on a large population-based survey of OOH-PC contacts comprising electronic registration of more than 4,000 OOH-PC telephone consultations during 12 months. Information on prescriptions was extracted from the patient administration system, and this procedure eliminated the risk of information bias. The integrated pop-up questionnaires, which appeared immediately after the completion of contacts, reduced the risk of recall bias. A former study has shown that the included contacts were highly representative of all contacts [12]. The formation of medication groups of antibiotics and analgesics across ATC-code groups optimised the clinical use of the data. Yet, the actual rate of using analgesics may be underestimated as we could not include advice on use of over-the-counter medication.

The categorisation of diagnosis groups was based on the probable diagnosis as stated in text by the GPs in the medical record in the patient administration system. The subsequent ICPC coding by the researchers was based on the exact wording of the diagnoses and may have induced some information bias as the text provided by the GPs could be ambiguous. However, the additional registered information on the contacts supported the choices of diagnosis.

We dichotomised each registered contact according to severity of the reported health problem (as assessed by the GPs) and excluded answers with ‘Don’t know’ from the analyses as we had no information on the reason for this answer. Consequently, 4.5% of all contacts were excluded, and this exclusion might have introduced a small risk of information bias.

### Interpretation

We found antibiotics to be the most frequently prescribed type of medication in the OOH-PC service, which is in line with other study results, although we focused on telephone consultations only [16,17]. The appropriateness of prescribing medication, in particular antibiotics, without seeing the patient may be questioned. Further research is needed to address this issue, especially when considering that the Danish organisation of the OOH-PC is based on having GPs in the front line to answer calls from patients and perform the triage.

Calls to the OOH-PC must be expected to be of an acute nature. We found that the frequency of prescriptions was significantly lower for ‘potentially severe’ than for ‘non-severe’ cases as assessed by the GPs. This finding is noteworthy as it could be expected that medical treatment was needed more often for severe problems. One of the explanations for this could be that the GPs in these cases assessed that immediate medical treatment was not needed and that the treatment was more optimally managed by the patients’ own GP in cases that were not acute, but gave rise to suspicion of potential severe disease. Conditions such as simple muscular-skeletal pain, migraine, and conjunctivitis account for many prescriptions in non-severe cases [18].

We found no differences between prescription rates for contacts due to new episodes and exacerbation of chronic disease. However, the share of analgesics prescribed was twice as high in contacts involving chronic disease. In contrast, the share of contacts involving antibiotic prescriptions was markedly higher in new episodes. Former studies have shown that GPs may feel pressed to start treatment [19,20]. Our study showed no association between prescribing behaviour and GP age, which may be due to the fact that telephone triage in Denmark is performed by fully trained GPs.

The direct telephone access to a GP may have a potential impact on the patients’ help-seeking behaviour, i.e. patients may find it more convenient or easier to call after office hours to get fast access to a GP. This hypothesis is supported by the finding that the prescription rate is higher during the first hours of the OOH primary care opening hours. Our results indicate a potential for optimising the use of GPs during daytime instead of the OOH-PC although the high prescription rates during daytime at weekends also highlight a need for prescription of medications in the OOH-PC.

## Conclusion

In one in five of all OOH-PC telephone consultations, medication was prescribed (most frequently antibiotics, analgesics, and respiratory-related medication). Patients aged 18-60 years had a higher probability of being prescribed medication, especially during weekends and within the first opening hours of the OOH-PC. The large number of prescriptions provided on the telephone demonstrates a pronounced need for looking more carefully into the demand for urgent medication prescriptions in the OOH-PC services and the appropriateness of the issued prescriptions; these issues should be addressed in future studies.

## Abbreviations

OOH-PC: Out-of-hours primary care; LV-KOS: Al survey on reasons for encounter and disease patterns in the Danish OOH-PC; ATC: The Anatomical therapeutic chemical classification system; ICPC-2: International classification of primary care (ICPC-2).

## Competing interests

The authors declare that they have no competing interests.

## Authors’ contributions

GM contributed substantially to the design, to acquiring, analyzing and interpretation of data, made the first draft and following revisions of the manuscript. LH gave substantial contribution to interpretation of data and participated actively in writing and revising the manuscript critically. MBC participated in the interpretation of data and in writing and revising the manuscript. PV contributed substantially to the design and interpretation of data and participated actively in writing and revising the manuscript. All authors read and approved the final manuscript.

## Pre-publication history

The pre-publication history for this paper can be accessed here:

http://www.biomedcentral.com/1471-2296/15/142/prepub

## Supplementary Material

Additional file 1 List of ATC codes for identifying antibiotic drugs. Click here for file

Additional file 2 List of ATC codes for identifying painkillers. Click here for file
