# Supplementary material for: Drug prescription by telephone consultation in Danish out-of-hours primary care: a population-based study of frequency and associations with clinical severity and diagnosis
Source: BMC Fam Pract. 2014 Aug 20;15:142. doi: 10.1186/1471-2296-15-142 (PMC4236596; doi:10.1186/1471-2296-15-142)
Supplement: Additional file 1 — List of ATC codes for identifying antibiotic drugs. [file 1471-2296-15-142-S1.pdf]

List of ATC codes for identifying antibiotic drugs

Category	ATC codes
D06A D07B, D07C, D07X	D06AX01<br>D06AX09<br>D06AX13<br>D06BX01<br>D06AA03<br>D07BC01<br>D07CA01<br>D07CC01
D06BA	D06BA01<br>J01XC01
G01	G01AF01<br>G01AA10
J01A	J01AA02<br>J01AA04<br>J01AA06<br>J01AA07<br>J01XE01
J01CA	J01CA01<br>J01CA02<br>J01CA04<br>J01CA08<br>J01CA11
J01CE	J01CE01<br>J01CE02
J01CF	J01CF01<br>J01CF05
J01CR	J01CR02
J01E	J01EA01<br>J01EB02
J01F	J01FA01<br>J01FA06<br>J01FA09<br>J01FA10<br>J01FF01
J01M	J01MA01<br>J01MA02<br>J01MA14
S01A og S02A	S01AB01<br>S01AX06<br>S01AX11<br>S01AX13<br>S01AX19<br>S01AX22<br>S01AA01<br>S01AA12<br>S01AA13<br>S01AA30

	S02AA15
S01C, S02C og S03C	S01CA01<br>S02CA02<br>S02CA03<br>S03CA01<br>S03CA04
J01DB, J01DC, J01DH og J01GB	J01DB01<br>J01DC02<br>J01DH03<br>J01GB01
