# Supplementary material for: Drug prescription by telephone consultation in Danish out-of-hours primary care: a population-based study of frequency and associations with clinical severity and diagnosis
Source: BMC Fam Pract. 2014 Aug 20;15:142. doi: 10.1186/1471-2296-15-142 (PMC4236596; doi:10.1186/1471-2296-15-142)
Supplement: Additional file 2 — List of ATC codes for identifying painkillers. [file 1471-2296-15-142-S2.pdf]

List of ATC codes for identifying painkillers

ATC codes
D04AB
M01AE
N02A
N02BE
R02AD
S01H
